# Finger millet-based muffin decreases insulin response in individuals with prediabetes in a randomised controlled trial

**DOI:** 10.1017/S0007114522001623

**Published:** 2023-02-28

**Authors:** Ameerah Almaski, Shelly Coe, Helen Lightowler, Miriam E Clegg, Pariyarath Sangeetha Thondre

**Affiliations:** 1 Department of Nutrition and Food Science, Taibah University, Universities Road, PO Box: 344, KSA, Medina, Saudi Arabia; 2 Oxford Brookes Centre for Nutrition and Health (OxBCNH), Department of Sport, Health Sciences & Social Work, Oxford Brookes University, Oxford, OX3 0BP, UK; 3 Department of Food and Nutritional Sciences, University of Reading, Whiteknights, Reading RG6 6AP, UK

**Keywords:** Finger millet, Prediabetes, Glycaemic response, Insulinaemic response, Gastric emptying

## Abstract

Millet is a grain high in polyphenols and antioxidants, which are bioactive compounds known to influence blood glucose response. The aim of this study was to compare the effect of finger millet muffin and wheat muffin on glycaemic response (GR), insulin response (IR), gastric emptying (GE) and satiety in healthy individuals and people with prediabetes. In a single-blind randomised controlled crossover trial at Oxford Brookes Centre for Nutrition and Health, fifteen healthy individuals and fourteen individuals with prediabetes were recruited between May and December 2017. The participants’ GR (3 h), IR (3 h), GE (4 h) and satiety (4 h) were measured before and after the consumption of muffins. A mixed method ANOVA was used to compare GE and the incremental AUC (iAUC) for GR and IR between the participant groups and muffins. There was a significant interaction between participants and muffins on IR iAUC at 180 min (*P* = 0·042). A significant effect of muffins was found on the GR peak (*P* = 0·013). The millet muffin decreased the GR peak and IR iAUC compared with the wheat muffin in participants with prediabetes. A significant interaction between participants and muffins for GE ascension time T_asc_ (*P* = 0·017) was observed, with no effect of muffins on satiety AUC in the participant groups. This study suggested that polyphenol and fibre-rich finger millet may have the potential to influence the management of prediabetes.

Prediabetes is defined as a condition where blood glucose levels are elevated above optimal levels but remain below the threshold required for clinical diagnosis of diabetes^(1)^. Each year, between 5 % and 10 % of individuals with prediabetes are shown to progress to clinical type 2 diabetes^(2)^. If poorly managed, prediabetes is linked to adverse cardiovascular outcomes. Unfortunately, there is insufficient awareness of this link among the general population with existing prediabetes^(3)^. Early diagnosis and treatment of individuals with prediabetes can not only delay but also prevent the development of type 2 diabetes and minimise the risk of consequent cardiovascular abnormalities^(3)^. According to Hsueh *et al.* ^(3)^, there are currently no approved medical treatments for prediabetes, with lifestyle changes remaining the only effective option.

Glycaemic response (GR) of a meal is the effect its consumption has on blood glucose levels, with the peak and duration of the increase dependent on the amount and type of carbohydrates ingested. Ensuring adequate regulation of postprandial blood glucose increase is especially crucial for people with prediabetes in order to reduce their risk of developing type 2 diabetes. Foods rich in polyphenols^(4)^ and dietary fibre^(5)^ are known to improve glucose tolerance and insulin sensitivity. Additionally, soluble dietary fibre decreases the rate of gastric emptying (GE)^(6)^, which is also responsible for maintaining a feeling of satiety and preventing glucose spikes.

Finger millet (*Eleusine coracana*) is a grain consumed by a large proportion of South Asian and African populations; its seed coat is rich in polyphenols, dietary fibre, vitamins and minerals^(7)^. In addition, the starch in finger millet is known to be slowly digested and assimilated, compared with that in other cereals^(8)^. Regular consumption of finger millet has been found to significantly lower peak plasma glucose and the AUC of glucose in patients with type 2 diabetes mellitus^(9,10)^. A number of studies have shown that a diet including finger millet is associated with lower blood glucose levels, compared with a rice-based diet, in patients with type 2 diabetes mellitus^(5,11)^. This may be a result of the high content of complex carbohydrates and resistant starch in finger millet, which leads to a slower rise in blood glucose^(11)^. It is not yet known whether millet-based products will exert an attenuating effect on blood glucose and insulin levels in individuals with prediabetes. Therefore, the aim of this study was to determine the effect of finger millet muffin consumption on postprandial GR, insulin response (IR), GE and satiety in healthy individuals and individuals with prediabetes. It is hypothesised that finger millet muffin consumption will lower the blood glucose levels in comparison with wheat muffins in individuals with prediabetes.

## Materials and methods

### Participants

Participant recruitment was done from May to December 2017. Two groups of participants were recruited for the study: healthy individuals and individuals with prediabetes.

#### Group 1

Sixteen healthy participants were eligible based on the following inclusion criteria: no known medical conditions, 18–65 years of age, BMI ≤ 30 kg/m^2^, fasting blood glucose level < 6·1 mmol/l, no known diabetes and no impairment in glucose tolerance.

#### Group 2

Fourteen participants were included in the prediabetes group. The inclusion criteria were 18–65 years of age, a BMI ≥ 25 kg/m^2^, a fasting blood glucose level of 6·1–6·9 mmol/l and/or 7·9–11·0 mmol/l 2 h after an oral glucose tolerance test. Certain factors such as being over 40 years of age, South Asian ethnicity, genetic factors (e.g. having a close relative diagnosed with diabetes) and a history of gestational diabetes and/or polycystic ovary syndrome (PCOS) may increase the risk of developing type 2 diabetes^(12)^. Volunteers with one or more of the above factors were invited for a screening, when they were subjected to a fasting blood glucose measurement and an oral glucose tolerance test, to assess their eligibility to participate. Participants were excluded if they were taking any medication that would affect their glucose regulation, GE, appetite or body weight. None of the participants were smokers.

Participants were recruited by the lead researcher (AA) through advertisements posted on university notice boards, supermarkets, libraries, community centres, diabetes and prediabetes support groups, surgery and health centre waiting rooms in Oxford, advertising sites (Gumtree and Daily Info) and the Diabetes UK website. Interested volunteers were invited to attend a screening session at Oxford Brookes Centre for Nutrition and Health, either before or on the morning of the test visit, where an information sheet was provided and a health questionnaire was completed, including details of smoking habits, food allergies/intolerances, metabolic diseases, physical activity, medication, eating habits and disorders. This study was conducted according to the guidelines laid down in the Declaration of Helsinki, and all procedures involving human subjects were approved by the University Research Ethics Committee (UREC) at Oxford Brookes University (UREC Registration No: 161061). Written informed consent was obtained from all subjects. The trial was registered on the ClinicalTrials. Gov registry (NCT04599738; https://clinicaltrials.gov/ct2/show/NCT04599738).

### Screening using oral glucose tolerance test

Fasting blood glucose, anthropometric measures and blood pressure were measured to confirm the eligibility of the participants for each group. Height was recorded using a stadiometer (Seca Ltd), with shoes removed. Body weight was recorded using a Tanita BC-418 MA scale (Tanita UK Ltd), with heavy garments and shoes removed.

The participants were asked to fast overnight (12 h) and then consume 75 g of dextrose (unflavoured, Myprotein) dissolved in 250 ml of water. Plasma glucose was measured before consumption of glucose and after 2 h using an automatic blood glucose analyser (Glucose 201+, Hemocue, Radiometer Ltd). The analyser was calibrated daily using control solutions (GlucoTrol-NG, Level 2, 1·0 ml) to ensure accuracy of results within a defined range. Prediabetes was diagnosed if oral glucose tolerance test blood glucose results after 2 h were in the range of 7·9–11·0 mmol/l^(13)^.

### Test food preparation

The test meals were two types of muffins provided on two different occasions: a control muffin (100 % wheat flour) and a finger millet muffin (50 % finger millet grain, 50 % wheat flour). Test meals were prepared in the Oxford Brookes Centre for Nutrition and Health kitchen by the lead researcher (AA). The finger millet muffins were prepared 1 d prior to testing, using a standardised recipe (consisting of 1 egg, 5 ml of vanilla extract, 125 ml of semi-skimmed milk, 32 g of caster sugar, 15 g of baking powder and 62 ml of sunflower oil) modified by replacing 50 % of wheat flour with crushed finger millet grain. Muffins were baked at 150°C for 25 min, cooled for 10 min and stored at room temperature until serving to the participants the following morning.

The serving size of muffins were determined based on the nutritional analysis carried out by Eurofins Food Testing UK Ltd (Wolverhampton, UK). Muffins were matched to contain 50 g of available carbohydrate per portion; control muffins (133·69 g) and finger millet-based muffins (145·77 g) were served with 250 ml of water. The control muffin had the following nutritional composition (per portion): energy: 1783 kJ; available carbohydrates: 50·0 g; total sugars: 12·4 g; crude protein: 8·4 g; total fat: 21 g and total dietary fibre: 1·7 g. The nutrient content of the finger millet muffin (per portion) was as follows: energy: 1831 kJ; available carbohydrates: 50·0 g; total sugars: 13·7 g; crude protein: 9·0 g; total fat: 21·0 g and total dietary fibre: 6·3 g.

### Analysis of total polyphenol content and antioxidant activity in the test foods

A 1-g sample of each muffin was extracted in amber bottles with 4 ml of solvent (70 % acetone) in a shaking water bath for 2 h at room temperature^(14)^. The extracts were centrifuged at 3000 × g for 10 min using a benchtop centrifuge (Heraeus Instruments, Kendro Laboratory Products, D-37520 Osterode). The supernatant was used to determine the antioxidant activity and total phenolic content. The extracts were covered with foil to protect them from light and frozen at –20°C until analysis.

### Total polyphenol content

To an aliquot of the muffin extract (200 μl), 1·5 ml of freshly prepared Folin–Ciocalteu reagent (1:10 v/v with water) was added. For 5 min, the mixture was allowed to equilibrate and then mixed with 1·5 ml of 60 g/l Na carbonate solution. After incubation of the mixture in the dark for 90 min at room temperature, the absorbance of the mixture was measured at 760 nm using 70 % acetone as blank. Gallic acid was used as a standard. The result was expressed as µg of gallic acid equivalents (GAEs) per gram of muffin samples^(15)^.

### Ferric ion reducing antioxidant power

A ferric ion reducing antioxidant power reagent was freshly prepared by mixing 300 mM of acetate buffer (pH 3·6), 20 mM of ferric chloride and 10 mM of tripyridyl-s-triazine made up in 40 mM of HCl, in the ratio 10:1:1, respectively. Ferrous sulphate (1000 μM) was used as a standard. One millilitre of purified water was taken in a tube and incubated at 37°C for 5 min. Twenty-five microlitre of muffin extract or standard was added to the tube with 1·0 ml of purified water, followed by the addition of 1 ml of ferric ion reducing antioxidant power reagent. To perform the assay, the mixture was again incubated at 37°C for 4 min and the absorbance was measured at 593 nm^(16)^.

### Study design

A single-blind randomised controlled crossover study design was used. Participants received each of the two muffins (wheat and finger millet) in random order at two separate study visits with at least a 1-d washout period between each visit. The muffin order was randomised using a pseudo-random number generator by the lead researcher (AA). During the 24 h prior to testing, participants were asked to avoid caffeine, alcohol, nicotine and non-routine strenuous exercise. All participants fasted for 12 h (overnight) and only consumed water during the fasting period.

### Sample collection procedures

#### Measurement of the glycaemic response

Blood for glucose and insulin tests was obtained using the finger-prick method and a single-use lancing system (Unistick 3, Owen Mumford) at −5 min (before consumption of muffin), 0 min (baseline) and 15, 30, 45, 60, 90, 120, 150 and 180 min post-consumption. Fasting blood samples were taken in duplicate (at −5 and 0 min) to ensure that the results were within a CV of 3 %. The first two drops of blood were discarded as they might have been contaminated and, therefore, may result in skewed values. The fingertips were then lightly massaged to extract blood from the base to the tip. The method used to measure GR was in line with FAO/WHO recommendations^(17)^.

#### Measurement of the insulinaemic response

For measuring insulin, blood samples were taken at the same time points and using the same finger-prick technique as for GR. At each time point, approximately 300 μl of blood was collected in EDTA-coated microtainer tubes (Bunzl Healthcare) and stored immediately on ice. All samples were centrifuged at 4000 rpm for 10 min, following which 200 μl of supernatant plasma was collected into Eppendorf tubes and frozen at −40°C, until analysis. Plasma samples were analysed for insulin using the electrochemiluminescence immunoassay and an automated analyser (Cobas® E411; Roche Diagnostics).

The changes in the GR and IR of participants after consumption of each muffin was calculated geometrically as the incremental AUC (iAUC, using the trapezoidal rule^(17)^, and only the area above the fasting level was included in the analysis.

#### Measurement of gastric emptying

GE was measured using 100 mg of ^13^C Na acetate added to the control and finger millet muffins^(18)^. Breath samples of participants were collected during each visit prior to muffin consumption (–5 and 0 min), then during the postprandial 4-h period at the same time points as for GR and IR – plus at 210 and 240 min. The samples were collected using a drinking straw by blowing into a 10-ml exetainer® tube (Labco), removing the straw, just prior to the end of exhalation. The cap of the tube was immediately replaced, and the samples were stored at room temperature for analysis. All participants were asked to wear a nose clip during sampling.

Breath samples were analysed using GC isotope ratio mass spectrometry (ABCA, SerCon Limited), and all results were expressed relative to Vienna PeeDee Belemnite (V-PDB), an international standard of known ^13^C composition. Data was expressed as % of ^13^CO_2_ dose recovered per hour and cumulative % of ^13^CO_2_ recovered over time. The production of CO_2_ was assumed as 300 mmol/m^2^ of body surface area per hour. The body surface area was calculated using a pre-validated weight–height formula^(19)^, which was then fitted into a GE model developed by Ghoos *et al.* ^(20)^. From this model, the lag phase, latency phase, ascension time and half time were then calculated. Lag phase (T_lag_) is the time taken from test food ingestion to maximal rate of ^13^CO_2_ excretion. Half time (T_half_) is the time it takes for 50 % of the ^13^C dose to be excreted. Latency phase (T_lat_) is the initial delay in the excretion curve. Ascension time (T_asc_) is the time between T_lag_ and T_half_ representing a period of high ^13^CO_2_ excretion rates^(21)^.

#### Measurement of hunger and satiety

A 7-anchor bidirectional scale was used to assess the satiety of participants at 0, 15, 30, 45, 60, 90, 120, 150, 180, 210 and 240 min time points, during each visit, immediately prior to blood sampling. The AUC was calculated geometrically using baseline scores as a covariate, as it corrects for baseline differences when analysing appetite scales^(22)^. The rating of the scale was as follows: 6 = extremely full and 0 = extremely hungry.

### Statistical analysis

Statistical analysis was undertaken using the Statistical Package for the Social Sciences (IBM SPSS, v. 25). The data on total polyphenol content and antioxidant activity of muffins, participant characteristics, satiety AUC, GE curves and change in blood glucose and plasma insulin are reported as mean and standard devation to show the variability in the data^(23)^. The results for glucose and insulin iAUC, GR and IR peak and time of peak and GE time are presented as mean with their standard errors. Before statistical analysis, the total polyphenol content and antioxidant activity of muffins, incremental data for the GR, IR and GE of muffins in each group (prediabetes and healthy) were tested for normality using a Shapiro–Wilk test.

The total polyphenol content and antioxidant activity of muffins were analysed using independent samples *t* test (normally distributed data) or Mann–Whitney *U* test (not normally distributed data). A two-way mixed method (muffin types × participant groups) ANOVA was used to compare GE, the iAUC for GR and IR between the participant groups and muffins. In all analyses, *P*-values < 0·05 were considered to be statistically significant. For the satiety AUC, a one-way ANCOVA was carried out, using the baseline as a covariate, to determine differences between muffin types. This was to avoid errors caused by using iAUC derived by simple subtraction of baseline values^(22)^. Previous statistical power calculations^(24)^ demonstrated that in order to achieve a power of 0·9, a total sample size of 12 was required, based on the difference in mean GR-iAUC of 10 mmol/l, with a mean sd of 5 mmol/l and an *α* of 0·05.

## Results

### Total polyphenol content and antioxidant activity of the test foods

The finger millet muffin had significantly higher total polyphenol content compared with the control muffin (572 ± 38 µg/g GAE *v*. 277 ± 27 µg/g GAE; *P* < 0·001). The total antioxidant activity of finger millet muffin was also significantly higher than the control muffin (602·91 ± 34·36 µmol/g *v*. 444·22 ± 8·94 µmol/g; *P* = 0·001).

### Participant characteristics

**Fig. 1.**
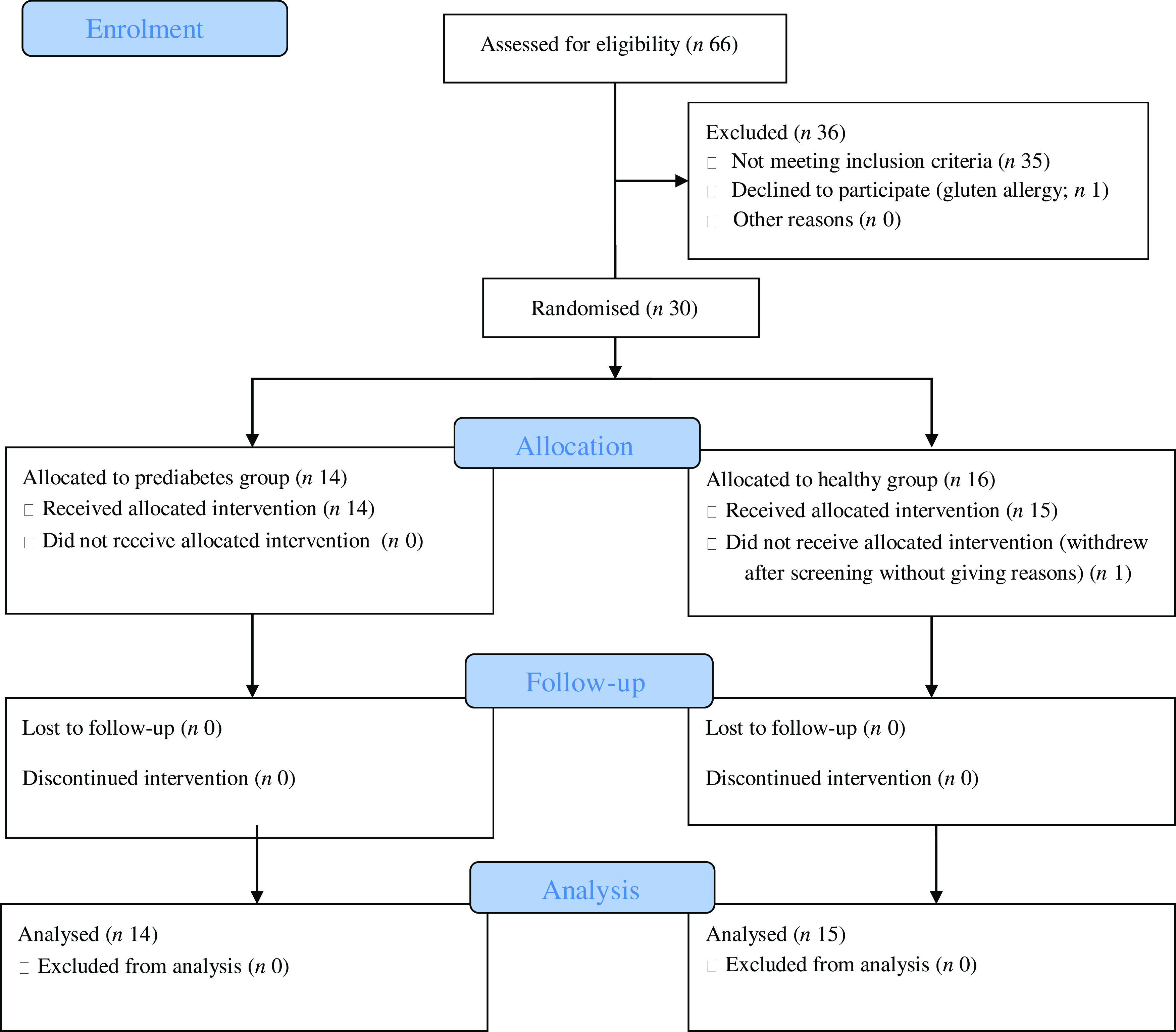
Consort flow diagram.

Sixteen healthy individuals were recruited and fifteen completed the study; one participant withdrew after the screening session without giving reasons (Fig. 1). Fifty people who had risk factors for prediabetes attended the screening sessions and fourteen completed the study. Thirty-five volunteers did not meet the inclusion criteria and one volunteer did not participate due to gluten allergy. There were no adverse events in the study. Demographic characteristics showed that there were no significant differences in age, systolic blood pressure or height between the two groups (Table 1). However, weight, BMI and diastolic blood pressure were significantly higher in the prediabetes group (*P* < 0·05).

**Table 1.** Demographics of participants with prediabetes and healthy individuals participating in the randomised controlled trial consuming wheat muffin and finger millet muffin (Mean values and standard deviations)

Characteristic	Prediabetes volunteers, *n* 14		Healthy volunteers, *n* 15		*P*
	Mean	sd	Mean	sd	
Age (years)	44·6	10·7	38·6	14·5	0·215
Sex	9 (female)/5 (male)		9 (female)/6 (male)		0·815
BMI (kg/m^2^)	31·7	3·1	24·6	2·9	<0·001*
Height (cm)	167·8	10·9	169·1	8·5	0·054
Weight (kg)	89·7	14·7	70·9	14·1	0·002*
Systolic BP (mm Hg)	119·9	16·1	111	11·8	0·063
Diastolic BP (mm Hg)	82·2	6·4	73·1	7·1	0·001*

BP, blood pressure.

* Significance level *P* < 0·05 for comparison between both groups.

### Glycaemic response and insulin response

**Fig. 2.**
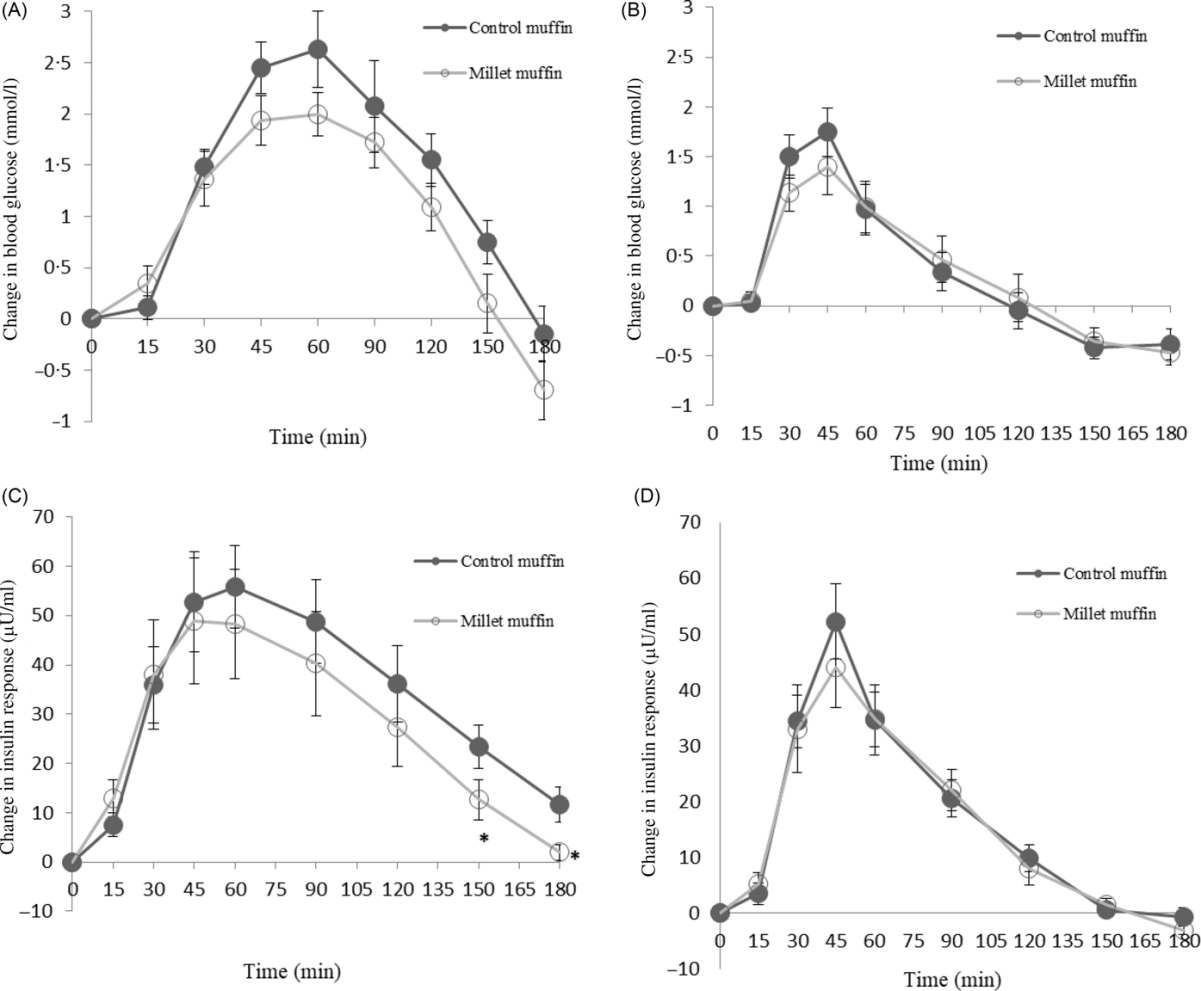
Changes in the blood glucose response from baseline (mmol/l) after wheat (control) and finger millet muffin consumption in individuals with prediabetes (a) and healthy participants (b). Changes in the insulin response from baseline in individuals with prediabetes (c) and healthy participants (d). Data are given as mean and standard devation. *Significant difference *P* < 0·05 compared with wheat muffins.

The incremental blood glucose concentrations in people with prediabetes and healthy individuals are depicted in Fig. 2(a) and (b), respectively. There was no significant effect of muffins on GR iAUC at 60 min, 90 min, 120 min, 150 min or 180 min (*P* > 0·05). However, there was a significant effect of participant groups on GR iAUC at 60 min (*P* = 0·036), 90 min (*P* = 0·001), 120 min (*P* = 0·001), 150 min (*P* = 0·002) and 180 min (*P* = 0·035) demonstrating a higher response in participants with prediabetes (Table 2a). There were no significant interactions between participant groups and muffins at 60 min, 90 min, 120 min, 150 min or 180 min (*P* > 0·05).

**Table 2a.** GR and IR iAUC for individuals with prediabetes and healthy participants after finger millet and wheat muffin consumption (Mean values with their standard errors)

		GR iAUC (mmol/l.min)						IR iAUC (μU/ml/min)				
iAUC (min)	Participant groups	*P*	Wheat muffin		Millet muffin		Participant groups	*P*	Wheat muffin		Millet muffin	
			Mean	sem	Mean	sem			Mean	sem	Mean	sem
60	Prediabetes	0·036*	81·5	8·6	70·3	8·6	Prediabetes	0·88	1865·9	328	1860·5	451·5
	Healthy		58·1	8·3	48·9	8·3	Healthy		1623·0	192·5	1507·2	252·8
90	Prediabetes	0·001*	150·3	14·4	123·0	14·4	Prediabetes	0·314	3375·4	539·6	3089·6	696·8
	Healthy		79·6	13·9	74·0	13·9	Healthy		2417·9	287·4	2312·4	337·4
120	Prediabetes	0·001*	192·4	19·0	152·2	19·0	Prediabetes	0·081	4322·9	711·4	3722·2	848·9
	Healthy		89·6	18·4	87·5	18·4	Healthy		2588·6	298·4	2477·3	320·4
150	Prediabetes	0·002*	197·9	21·1	149·1	21·1	Prediabetes	0·006*	4551·3	762·5	3672·2	866·4
	Healthy		92·3	20·4	93·6	20·4	Healthy		2110·3	249	2067·2	246·7
180	Prediabetes	0·035*	175·4	21·1	128·7	21·1	Prediabetes	<0·001*	4264·9	711·2	3184·5	771·2
	Healthy		93·6	20·4	95·5	20·4	Healthy		1508·2	182·8	1510·0	186·2

GR, glycaemic response; IR, insulin response; iAUC, incremental AUC.

*P*-values show the main effect of participant groups on GR and IR iAUC. *P < 0.05.

The changes in the IR from baseline in people with prediabetes and healthy individuals are depicted in Fig. 2(c) and (d), respectively. There was no significant effect of muffins on IR iAUC at 60 min, 90 min, 120 min, 150 min or 180 min (*P* > 0·05). Similarly, there was no significant effect of participant groups on IR iAUC at 60 min, 90 min or 120 min (*P* > 0·05). However, a significant effect of participant groups on IR iAUC was detected at 150 min (*P* = 0·006) and 180 min (*P* < 0·001) demonstrating a lower response in healthy participants (Table 2a). There were no significant interactions between participant groups and muffins at 60 min, 90 min, 120 min or 150 min (*P* > 0·05). However, there was a significant interaction between participants and muffins for the IR (iAUC) at 180 min (*P* = 0·042) demonstrating a significant difference in IR by muffin type in the prediabetes group alone. Compared with the control muffin, the IR was significantly lower following the millet muffin in the prediabetes group (Fig. 2(c)).

The differences in peak values and time to peak for GR and IR in the prediabetes and healthy groups after finger millet and control muffin consumption are depicted in Table 2b. There was a significant effect of muffins on the GR peak (*P* = 0·013) with the millet muffins generating a lower peak value. This is demonstrated in Fig. 2(a) where the peak glucose level was attenuated following the finger millet muffin consumption in the prediabetes group. However, there was no significant effect of muffins on the IR peak (*P* = 0·080). There was a significant effect of participant groups on the GR peak (*P* < 0·001) demonstrating a higher peak in participants with prediabetes. This significant effect of participant groups was not evident on IR peak (*P* = 0·269). There was no significant effect of muffins on GR time peak or IR time peak (*P* > 0·05). On the contrary, there was a significant effect of participant groups on GR time peak (*P* = 0·015) and IR time peak (*P* = 0·024) showing individuals with prediabetes taking longer time to reach peak response (Table 2b). There were no significant interactions between participants and muffins for the GR peak, the IR peak, the GR time to peak or the IR time to peak (*P* > 0·05).

**Table 2b.** Peak and time to peak values for individuals with prediabetes and healthy participants after finger millet and wheat muffin consumption (Mean values with their standard errors)

	Participant group	*P*	Wheat muffin		Millet muffin	
			Mean	sem	Mean	sem
GR peak (mmol/l)	Prediabetes	<0·001*	9·1	0·3	8·5	0·2
	Healthy		7·1	0·3	6·7	0·2
GR time to peak (min)	Prediabetes	0·015*	56·3	4·7	62·7	8·8
	Healthy		40	2·4	48	3·9
IR peak (µU/ml)	Prediabetes	0·269	80·2	10·9	78·3	13·8
	Healthy		63·6	6·4	58·6	8·0
IR time to peak (min)	Prediabetes	0·024*	67·5	6·5	72·9	10·5
	Healthy		45	2·1	51	3·8

GR, glycaemic response; IR, insulin response.

*P*-values show the main effect of participant groups on GR and IR peak and time of peak. *P < 0.05.

### Gastric emptying

**Fig. 3.**
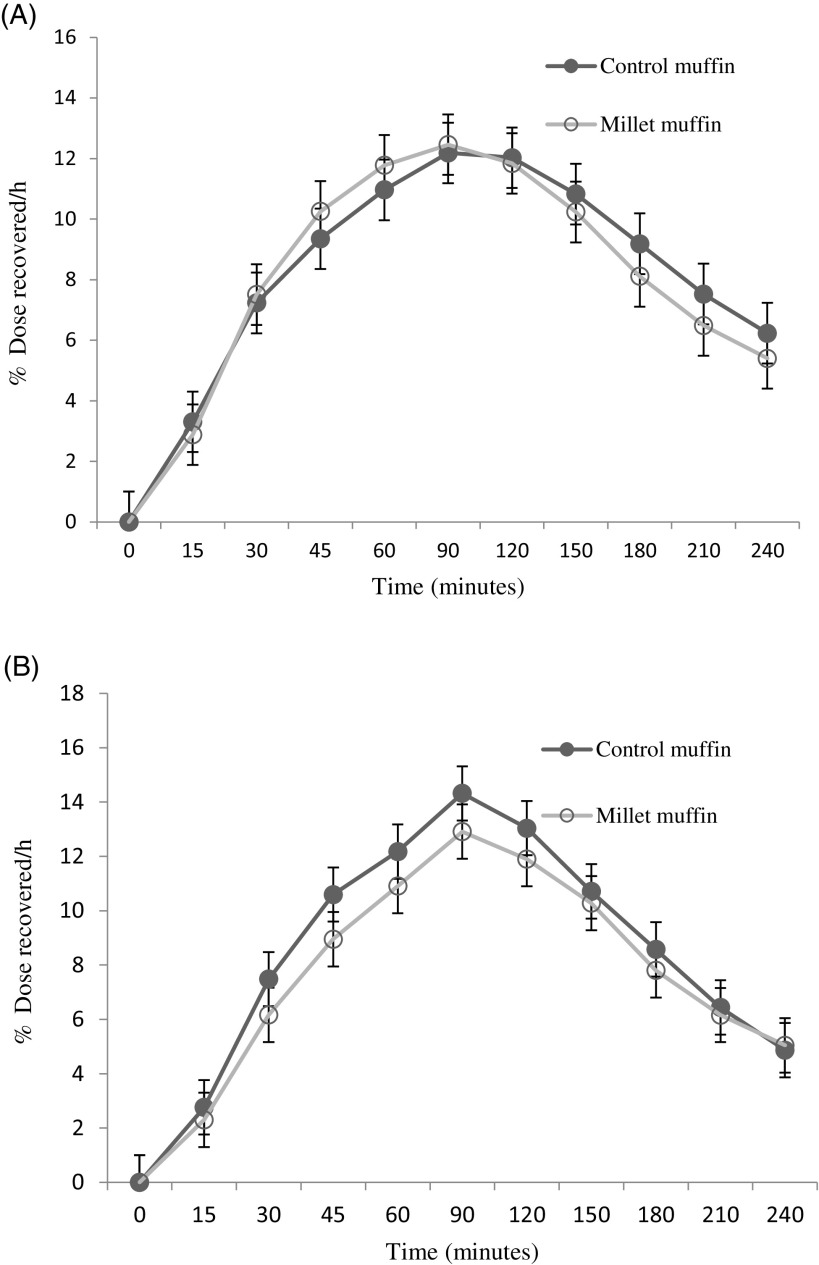
Breath ^13^CO_2_ curves following wheat (control) and finger millet muffin consumption for individuals with prediabetes (a) and healthy (b) participants. Data are presented as mean and standard deviation.

The differences in GE times between the control and finger millet muffins in people with prediabetes and healthy individuals in terms of T_half_, T_lag_, T_lat_ or T_asc_ are depicted in Fig. 3(a) and (b), respectively. There were no effects of muffins or participants on any of the GE parameters (Table 3). Similarly, there were no significant interactions between participants and muffins for T_half_, T_lag_ and T_lat_ (*P* > 0·05). However, a statistically significant interaction between participants and muffins for T_asc_ (*P* = 0·017) was observed suggesting that the finger millet muffin was emptying quickly compared with the control muffin in the prediabetes group.

**Table 3.** GE time after finger millet and wheat muffin consumption in individuals with prediabetes and healthy participants (Mean values with their standard errors)

GE time (min)	Participant group	*P*	Wheat muffin		Millet muffin	
			Mean	sem	Mean	sem
T_half_	Prediabetes	0·689	82·02	14·9	61·40	9·4
	Healthy		55·56	4·4	62·40	5·6
T_lag_	Prediabetes	0·986	32·42	5·5	25·74	5·8
	Healthy		26·34	5·2	32·03	5·4
T_lat_	Prediabetes	0·614	37·25	4·7	35·41	4·5
	Healthy		36·18	4·4	41·56	4·1
T_asc_	Prediabetes	0·117	120·61	12·6	99·36	7·8
	Healthy		92·05	3·3	94·33	3·6

GE, gastric emptying.

*P*-values show the main effect of participant groups on GE parameters.

### Satiety

Mean hunger and fullness ratings at baseline, 0 min and after ingestion of control or finger millet muffins, in both healthy and prediabetes groups, are shown in Table 4. Satiety was not significantly affected (*P* > 0·05) by either muffin type (finger millet or control) or participant group (healthy or prediabetes).

**Table 4.** AUC for satiety following wheat and finger millet muffin consumption in individuals with prediabetes and healthy participants (Mean values and standard deviations)

AUC (time in min.)	Participant group	Wheat muffin		Millet muffin		*P*
		Mean	sd	Mean	sd	
60	Prediabetes	267·3	85·1	287·7	64·8	0·4
	Healthy	211·5	68·9	223	71·6	0·588
90	Prediabetes	389·5	131·3	428·0	91·1	0·311
	Healthy	300·5	105·1	327	111·0	0·4
120	Prediabetes	497·7	180·4	565·2	128·5	0·211
	Healthy	377·5	131·5	423	148·9	0·257
150	Prediabetes	598·4	225·2	673·4	175·2	0·288
	Healthy	439·5	156·9	497·5	181·7	0·193
180	Prediabetes	689·5	257·8	735·5	216·2	0·582
	Healthy	490·5	180·1	524·5	197·4	0·329
210	Prediabetes	767·7	282·8	765·0	249·4	0·977
	Healthy	531·5	198·5	517	203·1	0·737
240	Prediabetes	820·2	301·1	772·0	280·6	0·647
	Healthy	556·0	216·6	499	210·8	0·86

## Discussion

The main findings of this study were that finger millet muffin consumption resulted in a significantly attenuated postprandial IR and peak blood glucose level in participants with prediabetes. However, the reduction in GR was not statistically significant after finger millet consumption. No change was observed in GE or satiety in the prediabetes or healthy groups after finger millet consumption

The attenuated IR after finger millet muffin consumption is in agreement with Abdelgadir *et al.* ^(25)^, who reported a significant decrease in the insulin AUC after proso millet porridge consumption in comparison with sorghum and maize porridge in patients with type 2 diabetes mellitus. Fibre and polyphenol contents of finger millet may be responsible for the decrease in IR. Finger millet is higher in dietary fibre compared with other cereals^(26)^, and previous randomised controlled trials have shown that fibre-fortified foods significantly decrease postprandial GR and IR^(27,28)^. The total fibre content of finger millet muffin used in this study was almost four times that of the control (wheat) muffin, suggesting the possibility that dietary fibre played a role in the reduction in IR. The role of non-starch polysaccharides and resistant starch in delaying nutrient digestion and absorption has been extensively reported in a review on health benefits of finger millet^(26)^

Finger millet is also high in polyphenols^(8)^, which may have a favourable effect on postprandial GR^(29)^ through inhibiting amylase and *α*-glucosidase enzymes and delaying glucose absorption^(26)^. Higher polyphenol content has been reported in the grains^(30)^ used to prepare the finger millet muffins, which had twice the amount of polyphenols compared with the wheat muffins in this study. A previous study^(31)^ using cinnamon suggested that insulin receptor stimulation by polyphenols might augment glucose uptake and decrease insulin need, which may partly explain the attenuated IR. The GR and IR profiles of participants with prediabetes in this study indicate that the glucose uptake rate following finger millet muffin consumption may not have been sufficient to cause a significant lowering of GR. Yet, the demand for insulin release from pancreas may have been reduced towards the end of the 3-h postprandial period resulting in a significant reduction in IR. Polyphenols may also prevent increases in insulin in the early stage of prediabetes by preventing oxidative damage to *β*-cells and activating glucose uptake receptors in insulin-sensitive tissues, precluding any compensatory rise in insulin secretion as demonstrated by Paquette et al^(4)^. Whilst fibre and polyphenols are expected to cumulatively lower the GR iAUC of foods, soluble fibre has been reported to reduce the *in vitro* inhibitory activity of *α*-amylase by polyphenols^(32)^, which may explain the non-significant results of GR in this study

Cooking duration and heat intensity may influence the polyphenols and the ratio of the different starch fractions in millet, which ultimately may differentially affect the GR^(8)^. Thermogenic breakdown of phenolic molecules as well as decarboxylation and polymerisation of free phenolic acids are reported following cooking^(33)^. In this study, the baking temperature and duration of muffin preparation (150°C for 25 min) were more than those used for preparing roti (toasted and processed with oil) and dumplings (boiled) in previous studies^(8,11,34)^. A long cooking time could promote breakdown of starch granules, thereby increasing their surface area for rapid digestion and leading to an increase in the GR^(8)^. These structural changes by cooking might have influenced the GR and IR in this study. The effect of cooking on polyphenols’ properties warrant further research.

As expected, participants with prediabetes exhibited a significantly elevated GR iAUC compared with healthy individuals for both control and millet muffins. However, in contrast with previous studies^(8,11)^ where a reduction in GR was achieved in both healthy individuals and patients with type 2 diabetes mellitus after consumption of flatbread (*roti*), pancake (*dosa*) and dumplings prepared from whole finger millet, there was no significant participant × test meal interaction in this study alluding to possible differences in insulin secretion between prediabetes and type 2 diabetes^(1)^.

In the current study, there was no delay in GE in both the prediabetes and healthy groups after finger millet consumption. Cisse *et al.* ^(35)^ observed slower GE rates in healthy participants after millet consumption in solid and liquid food (couscous and porridge), compared with white rice, plain potato and wheat pasta. This may be explained by the larger portions of pure millet test foods (650 g) used by Cisse *et al.* ^(35)^ compared with smaller portion size of 146 g in this study (50 % of wheat flour replaced with about 36 g of finger millet grain to prepare the muffins). Moreover, as GE is delayed in patients with type 2 diabetes mellitus in response to hyperglycaemia^(36)^, a similar response may be observed in prediabetes. The significant delay in ascension time observed for control muffins compared with the millet muffins may suggest that the finger millet grains became separated from the muffin mixture in the stomach causing faster emptying in participants with prediabetes. GE is known to depend on glycaemic control, and glycaemic control in turn is influenced by GE^(37)^. This bidirectional relationship may be more pronounced in hyperglycaemia as seen in prediabetes, thereby promoting glucagon-like peptide-1 and insulin secretion^(37)^.

GE is directly linked to satiety^(6)^. Factors such as particle characteristics and water content of the foods affect GE^(38,39)^. The coarsely crushed form of millet may not have released the soluble fibre from the grain to delay GE and increase the feeling of satiety in this study. There was no relation between gastric volume measured by MRI and measures of satiety in a study following finger millet porridge consumption in healthy participants^(40)^. Pearl millet foods have also shown mixed results depending on particle characteristics^(38,39)^. Whilst a positive correlation between gastric volume and satiety measures was found with the swelling effect of millet flake porridge^(38)^, no effect on GE was observed with the sieving effect of millet flour porridge^(39)^.

To our knowledge, this is the first study to examine the effect of finger millet-based muffin on GR, IR, GE and satiety in people with prediabetes. A strength of the study is that the fibre and polyphenol content was known in the muffins used in this study. Moreover, other polyphenol-containing ingredients (e.g. vegetables and spices) were not used as in previous studies^(8,11)^, which might have interfered with the results. A larger sample size was used in this study compared with some of the previous studies on patients with type 2 diabetes mellitus^(8,11)^. A limitation is that only one dose of millet grains (36 g) and one type of food (muffins) were tested in this study. The dose of millet grains required to attenuate GR and IR may vary in other test foods depending on the ingredients present and the processing methods used. Therefore, the results in this study cannot be generalised to other test foods. Moreover, satiety was measured in this study using a subjective rating scale without measuring food intake at an *ad libitum* lunch. More robust and objective measurement of gastrointestinal hormones that reflect satiety, such as leptin and ghrelin, may be desirable to elucidate the effect of millet consumption on satiety^(41)^.

### Conclusion

The consumption of finger millet muffin resulted in lower glucose peak level and improved IR in individuals with prediabetes. Fibre and polyphenols in finger millet muffin may be contributing to the decrease in postprandial IR in people with prediabetes. Therefore, finger millet may be beneficial in dietary interventions to prevent the progression of prediabetes to type 2 diabetes. Future work is required to examine the effects of different doses of finger millet and different cooking methods on GR, IR, GE and satiety to maximise its nutritional benefits for people with prediabetes.
